# Association of Short-Term Heart Rate Variability With Breast Tumor Stage

**DOI:** 10.3389/fphys.2021.678428

**Published:** 2021-09-10

**Authors:** Shuang Wu, Man Chen, Jingfeng Wang, Bo Shi, Yufu Zhou

**Affiliations:** ^1^Department of Radiation Oncology, First Affiliated Hospital, Bengbu Medical College, Bengbu, China; ^2^School of Medical Imaging, Bengbu Medical College, Bengbu, China; ^3^Anhui Key Laboratory of Computational Medicine and Intelligent Health, Bengbu Medical College, Bengbu, China

**Keywords:** autonomic modulation, breast tumors, heart rate variability, nonlinear dynamics, tumor-node-metastasis stages

## Abstract

Cardiac autonomic modulation, assessed by heart rate variability (HRV), is associated with tumor pathogenesis and development as well as invasion and metastasis. This study aimed to examine this association in breast cancer (BC) patients. A total of 133 patients (average age 49.2years) with BC or benign breast tumors were divided into three groups: benign group, early-stage group, and advanced-stage group. About 5-min resting ECG was collected for the analysis of linear and nonlinear HRV parameters. Multiple logistic regression models were performed to test the independent contribution of HRV to breast tumor stage. The advanced-stage group had significantly reduced HRV compared to the benign and early-stage groups. In particular, for each 1-SD increase in SD2, SD of normal-to-normal intervals, very-low frequency, total power, and low frequency, the odds of having advanced staging decreased by 69.3, 64.3, 58.3, 53.3, and 65.9%, respectively. These associations were independent of age, body mass index, mean heart rate (HR), and respiratory rate (RR). These findings suggest an association between HRV and breast tumor stage, and HRV parameters may help construct an effective early diagnostic and clinical prognostic model.

## Introduction

Breast cancer (BC) is the most common cancer among women worldwide. In 2020, approximately 2.26 million new cases of BC were diagnosed. BC represents approximately 11.7% of all cancers and is the fifth leading cause of cancer deaths worldwide (680,000 deaths; Sung et al., 2021).

The vagus nerve, a major part of the parasympathetic nerve system, regulates the development and progression of cancer (De Couck and Gidron, 2013). Increased vagal nerve activity has a profound inhibitory effect on oxidative stress, DNA cell damage, inflammation, and sympathetic nervous system overreaction (Maki et al., 2006; Tsutsumi et al., 2007; Tracey, 2009; De Couck et al., 2012). Studies have demonstrated the bidirectional relationship between cancer and decreased vagal nerve activity (De Couck et al., 2018). Therefore, study of the vagus nerve could provide valuable prognostic information and guide therapy in breast cancer.

Heart rate variability (HRV) is a promising biomarker that can be used to evaluate autonomic nervous system function and it may be associated with vagal nerve function (Vanderlei et al., 2009; Karvinen et al., 2013). One group showed the clinical significance of HRV in patients with BC (Arab et al., 2016). However, few studies have explored the association of HRV with tumor-node-metastasis (TNM) in patients with BC.

In a previous study, patients with BC had lower the SD of all normal-to-normal intervals (SDNN) and the root mean square of successive interval differences (RMSSD) than women without BC, no matter how long after the surgery (Palma et al., 2016). In addition, Arab et al. (2018) found that SDNN and RMSSD negatively correlated with BC staging after analyzing time and frequency domains of HRV in patients. In patients with advanced-stage BC, low SDNN might be correlated with a poor prognosis. Kim et al. (2010) reported that an SDNN value of <21.3ms in brain metastasis predicted poor survival, while Wang et al. (2013) found that an SDNN value of <10ms in terminal-stage cancer predicted poor survival. These studies suggested a potential role of time-domain parameters of HRV as a prognostic factor. However, further research is warranted to clarify the potential role of time-domain parameters in the survival of patients with advanced-stage BC.

Previous studies indicated that higher resting high-frequency power (HF) was strongly associated with longer overall survival in patients with recurrent or metastatic BC (Giese-Davis et al., 2015). Chiang et al. (2010) found that the survival time of patients with terminal hepatocellular carcinoma was significantly related to HF. These studies suggested that, as a long-term predictor of survivors, HF may offer early estimation of clinical prognosis for cancer patients. More importantly, the results of these studies also indicated that HF strongly positively correlated with prognosis, particularly in patients with advanced-stage cancer. The vagal nerve activity might be of more importance in advanced stages.

Therefore, this study aimed to compare short-term HRV in the tumor stage of patients with BC. It was hypothesized that HRV in patients with advanced cancer would be lower than that in patients with early-stage disease. The analysis confirmed the aforementioned hypothesis and had clinical implications: HRV might be a potentially feasible tool in clinic to evaluate the prognosis of BC. More importantly, clinicians could ascertain patients at risk for disease progression through the long-term monitoring of HRV.

## Materials and Methods

### Participants and Procedures

This study followed the regulations of the National Research Ethics Committee and obtained the approval of the Clinical Medical Research Ethics Committee of the First Affiliated Hospital of Bengbu Medical College (Bengbu, Anhui, China; registration number: 2019KY031). In this study, women diagnosed with breast tumors were selected by the pathological examination from 2019 to 2020. All participants volunteered for this study and provided informed consent.

The function of the vagal nervous system was assessed by analyzing HRV using an ECG recorder (HeaLink-R211B; HeaLink Ltd., Bengbu, China). The sampling rate of the ECG signal was 400Hz. ECG data were collected at room temperature (23±1°C) and always 3days prior to radiotherapy/operation. Participants were explained the ECG collection procedure and were asked to assume the supine position and keep quiet during ECG examination. The ECG acquisition time using V5-lead was 5min.

The following conditions that are known to alter HRV were used as exclusion criteria: (1) diabetes mellitus; (2) heart diseases; (3) use of anti-arrhythmic drugs or beta-blockers; (4) pacemaker; (5) poor ECG quality; (6) ectopic beats (>10% of all beats); and (7) chemotherapy or surgery in the 3weeks before the examination. Therefore, our study analyzed the data of 133 participants.

### HRV Analysis

The ECG R peaks were extracted using an algorithm based on the Pan–Tompkins algorithm (Pan and Tompkins, 1985). The technical and physiological artifacts within R-R intervals (RRI) were corrected by applying an automatic artefact correction algorithm. Subsequent HRV indices for both linear (time and frequency domain) and nonlinear methods were calculated.

Commonly used time-domain indices include SDNN and RMSSD. SDNN, a total variability index, represents the involvement of all cyclic components. RMSSD reflects parasympathetic activity (Camm et al., 1996; Vanderlei et al., 2009).

The RRI time series was converted into power spectral analysis to analyze the frequency domain, and the power spectral density was obtained using the Fast Fourier Transform algorithm. Prior to frequency-domain analysis, the RRI time series was evenly resampled at 4Hz using cubic spline interpolation. Frequency-domain parameters included total power (TP, 0–0.4Hz), high-frequency power (HF, 0.15–0.4Hz), low-frequency power (LF, 0.04–0.15Hz), very-low frequency power (VLF, 0–0.04Hz), and the ratio of LF to HF (LF/HF). LF and HF parameters were expressed in normalized units: normalized HF [HF n.u. =HF/(TP – VLF)] and normalized LF [LF n.u. =LF/(TP – VLF); Montano et al., 1994; Camm et al., 1996; Vanderlei et al., 2009].

The LF corresponds to the co-regulation of sympathetic and vagal nerve tones, HF indicates the vagal nerve tone, and the LF/HF reflects interactions of both sympathetic nervous system and parasympathetic nervous system, but they are limited to the case where the respiratory frequency is in the HF band (Hernando et al., 2016; Varon et al., 2019). The difference in respiratory rate (RR) will lead to the analysis of HRV in the standard frequency band cannot accurately estimate the activity of autonomic nervous system. For example, when the RR is higher than the upper limit of the HF band, vagus activity may be underestimated. In contrast, when the RR is within the LF band, sympathetic activity is overestimated and vagus activity is underestimated. Therefore, respiratory influences need to be separated in order to better estimate the activity of sympathetic and vagus nerves (Varon et al., 2019). An estimate of RR was calculated using an ECG-derived respiration approach (Moody et al., 1985). It is important to check whether RR is below 0.15Hz or higher than 0.4Hz in all the enrolled subjects, in order to trust in the interpretation of LF and HF related indices.

Each RRI time series included eight nonlinear HRV indices, including approximate entropy (ApEn), sample entropy (SampEn), Poincare plot: SD1, SD2, and SD2/SD1, detrended fluctuation analysis (DFA): α1 and α2, and correlation dimension (CD). The estimated ApEn and SampEn depended on three parameters: the embedding dimension *m*, the tolerance value *r*, and the data length *N*. The parameters are set as *m*=2 and *r*=0.2*σ*, where *σ* was the SD of each realization (Camm et al., 1996; Vanderlei et al., 2009; Voss et al., 2009; de Godoy, 2016).

All the above processing steps were performed using the Kubios HRV Premium software (version 3.1.0, Kubios Oy, Kuopio, Finland).^1^

### Breast Tumor Groups

Patients with breast tumors were divided into three groups: benign group, early-stage group, and advanced-stage group. Patients with benign breast tumors (i.e., benign epithelial proliferations, intraductal papilloma, phyllodes tumor, breast hyperplasia, and fibroadenomas) were selected as the controls. According to the National Comprehensive Cancer Network Clinical Practice Guidelines TNM staging version 3.2020 (Gradishar et al., 2020), the remaining participants were divided into early-stage and advanced-stage groups. The early-stage group consisted of T1–2, N0–1, and M0, and T3N0M0 cancers, while the advanced-stage group consisted of T0–4, any N, and M0–1 cancers. The non-advanced-stage group included controls and early-stage patients.

### Statistical Analysis

Descriptive statistical data were expressed as mean (SD), median (Q1, Q3), or percentage. The Shapiro–Wilk test was used to test the normality of HRV indices. A chi-square test was used to analyze the difference between the two cancer groups. Dependent variable analyses for linear and nonlinear HRV parameters were separately conducted using parametric and nonparametric tests. One-way ANOVA was used to calculate normal data and Fisher’s least significant difference (LSD) was used for multiple comparisons between groups. The Kruskal–Wallis test was used to analyze non-normal data. Finally, separate multiple logistic regression models were performed with breast tumor stage as an outcome and with each significant HRV parameter set as a predictor while adjusted for age, body mass index (BMI), mean heart rate (HR), and RR. SPSS Statistics 25.0 (IBM Corp., Chicago, Illinois, United States) was used, and a value of *p*<0.05 was considered statistically significant.

## Results

Table 1 presents the demographics and HRV indices of patients with breast tumors. In TNM staging, the early-stage group mainly comprised patients with stages T1–2, N0–1, and M0 cancers, while the advanced-stage group commonly comprised patients with stages T1–3, N2–3, and M0 cancers. Twelve patients in the advanced-stage group had distant metastases to the brain, bones, and lungs. Among patients with stages I–IV, the early-stage group mostly comprised stages Ia, IIa, and IIb; however, the advanced-stage group mostly comprised stage IIIa, IIIc, and IV. Invasive carcinoma with no special type is mostly frequent in patients with BC. The early-stage group had a more noninvasive type of BC compared to the advanced-stage group. The results of the molecular typing revealed no significant differences between the groups (Table 2).

**Table 1:** Demographics and heart rate variability (HRV) of breast tumor patients.

Variables	Values
*N* (Female)	133
Age (years)	49.2 (10.5)
BMI (kg/m^2^)	24.5 (3.6)
Mean HR (bpm)	79.8 (11.5)
RR (Hz)	0.31 (0.05)
SDNN (ms)	28.8 (11.5)
RMSSD (ms)	17.1 (10.3, 24.1)
VLF (ms^2^)	359 (167, 573)
LF (ms^2^)	116 (59, 260)
HF (ms^2^)	122 (55, 255)
TP (ms^2^)	640 (344, 1,072)
LF n.u. (%)	53.1 (18.7)
HF n.u. (%)	47.1 (18.7)
LF/HF	1.102 (0.599, 2.114)
SD1 (ms)	12.1 (7.3, 16.9)
SD2 (ms)	38.1 (14.8)
SD2/SD1	3.392 (1.270)
ApEn	1.126 (0.091)
SampEn	1.441 (0.268)
α_1_	1.068 (0.263)
α_2_	1.070 (0.188)
CD	0.494 (0.228, 0.974)

ApEn, approximate entropy; BMI, body mass index; bpm, beats per minute; CD, correlation dimension; RR, respiration rate; HR, heart rate; HF, high-frequency power; LF, low-frequency power; LF/HF, ratio of low-frequency power to high-frequency power; N, number of individuals; RMSSD, root mean square of successive interval differences; SampEn, sample entropy; SD, standard deviation; SDNN, SD of all normal-to-normal intervals; TP, total power; and VLF, very low frequency. Values are expressed as the number of patients, mean (SD), or median (Q1, Q3).

**Table 2:** Clinical characteristics of breast cancer (BC) patients.

	Early stage (*N*=50) (%)	Advanced stage (*N*=40) (%)	*p*
TNM staging			
T – primary tumor in greatest dimension			
T1 (≤20mm)	20 (40)	11 (27.5)	**0.002**
T2 (>20mm and≤50)	28 (56)	15 (37.5)	
T3 (>50mm)	2 (4)	9 (22.5)	
T4 (any size with extension to the chest wall and/or to the skin – ulceration or skin nodules)	0 (0)	5 (12.5)	
N – regional lymph nodes metastases			
N0 (none)	35 (70)	0 (0)	**<0.001**
N1	15 (30)	5 (12.5)	
N2	0 (0)	16 (40)	
N3	0 (0)	19 (47.5)	
M – distant metastases			
M0 (no clinical or radiographic evidence)	50 (100)	28 (70)	**<0.001**
M1 (with distant detectable metastases)	0 (0)	12 (30)	
I–IV staging			
Ia – T1, N0, M0	16 (32)	0 (0)	**<0.001**
Ib – T0–1, N1 micrometastases, M0	0 (0)	0 (0)	
IIa – T0–1, N1, M0 or T2, N0, M0	21 (42)	0 (0)	
IIb – T2, N1, M0 or T3, N0, M0	13 (26)	0 (0)	
IIIa – T0–2, N2, M0 or T3, N1–2, M0	0 (0)	13 (32.5)	
IIIb – T4, N0–2, M0	0 (0)	3 (7.5)	
IIIc – T0–4, N3, M0	0 (0)	12 (30)	
IV – T0–4, N0–3, M1	0 (0)	12 (30)	
Type of breast cancer			
Noninvasive type	11 (22)	3 (7.5)	0.169
Invasive (no special type)	38 (76)	36 (90)	
Invasive (special type)	1 (2)	1 (2.5)	
Molecular typing			
Luminal A	12 (24)	10 (25)	0.794
Luminal B	19 (38)	14 (35)	
Her-2 overexpressing	10 (20)	11 (27.5)	
Triple negative	9 (18)	5 (12.5)	

Values are expressed as the number of individuals (percentages). Bold values of *p* indicate statistical significance (*p*<0.05). Luminal A: ER/PR (+), HER2 (−), and ki-67 <14%. Luminal B: ER/PR (+), HER2 (−), and ki-67 ≥14%; ER/PR/HER2 (+), any level of ki-67. Her-2-overexpressing: ER/PR (−) and HER2 (+). Triple negative: ER/PR/HER2 (−).

Differences in SDNN (*p*<0.001), RMSSD (*p*=0.006), VLF (*p*<0.001), LF (*p*<0.001), HF (*p*=0.005), TP (*p*<0.001), SD1 (*p*=0.007), SD2 (*p*<0.001), and CD (*p*<0.001) between groups were shown using one-way ANOVA and Kruskal–Wallis test. Furthermore, SDNN, RMSSD, VLF, LF, HF, TP, SD1, SD2, and CD were significantly decreased in the advanced-stage group compared to the corresponding values in the benign and early-stage groups. However, differences in HRV indices between the benign and early-stage groups were not significantly different. Moreover, there were no statistically significant differences in the LF n.u, HF n.u, LF/HF, SD2/SD1, ApEn, SampEn, α_1_, and α_2_ among the groups (Table 3).

**Table 3:** Comparison of HRV parameters among the benign, early-stage, and advanced-stage groups.

Variables	Benign (*N*=43)	Early stage (*N*=50)	Advanced stage (*N*=40)	*p*
Mean HR (bpm)	78.9±11.7	77.9±10.5	83.0±12.2	0.099
RR (Hz)	0.30±0.04	0.31±0.05	0.31±0.06	0.489
SDNN (ms)	32.7±9.3	29.3±11.6	23.1±11.6^*^^,^ ^†^	**<0.001**
RMSSD (ms)	19.2 (13.8, 27.4)	18.6 (12.2, 27.8)	11.2 (8.0, 18.0)^*^^,^ ^†^	**0.006**
VLF (ms^2^)	443 (259, 674)	410 (177, 604)	199 (103, 355)^*^^,^ ^†^	**<0.001**
LF (ms^2^)	155 (102, 297)	154 (59, 295)	66 (37, 142)^*^^,^ ^†^	**<0.001**
HF (ms^2^)	151 (78, 300)	151 (55, 270)	60 (23, 136)^*^^,^ ^†^	**0.005**
TP (ms^2^)	903 (485, 1,147)	781 (429, 1,190)	351 (187, 619)^*^^,^ ^†^	**<0.001**
LF n.u. (%)	52.7±18.6	54.4±18.9	52.0±18.9	0.821
HF n.u. (%)	47.2±18.5	46.4±19.1	48.0±18.9	0.927
LF/HF	1.214 (0.682, 2.030)	1.401 (0.579, 2.213)	0.952 (0.597, 2.165)	0.852
SD1 (ms)	13.6 (9.8, 17.3)	13.1 (8.7, 19.7)	8.0 (5.7, 12.8)^*^^,^ ^†^	**0.007**
SD2 (ms)	43.8±12.7	39.6±14.8	30.2±13.8^*^^,^ ^†^	**<0.001**
SD2/SD1	3.444±1.237	3.348±1.358	3.391±1.220	0.937
ApEn	1.10±0.10	1.13±0.10	1.15±0.10	0.055
SampEn	1.40±0.27	1.47±0.27	1.46±0.26	0.411
α_1_	1.11±0.26	1.07±0.27	1.02±0.25	0.355
α_2_	1.05±0.19	1.07±0.18	1.09±0.19	0.557
CD	0.73 (0.39, 1.05)	0.56 (0.31, 1.69)	0.20 (0.08, 0.57)^*^^,^ ^†^	**<0.001**

ApEn, approximate entropy; bpm, beats per minute; CD, correlation dimension; HF, high-frequency power; HR, heart rate; LF, low-frequency power; LF/HF, ratio of low-frequency power to high-frequency power; RMSSD, root mean square of successive interval differences; RR, respiration rate; SampEn, sample entropy; SD, standard deviation; SDNN, standard deviation of all normal-to-normal intervals; TP, total power; and VLF, very low frequency. Values are expressed as mean (SD) with normal distribution or median (Q1, Q3) without normal distribution. Bold values of p indicate statistical significance (value of *p*<0.05).

* *p*<0.05 advanced-stage group vs. benign group.

† *p*<0.05 advanced-stage group vs. early-stage group.

**Figure 1:**
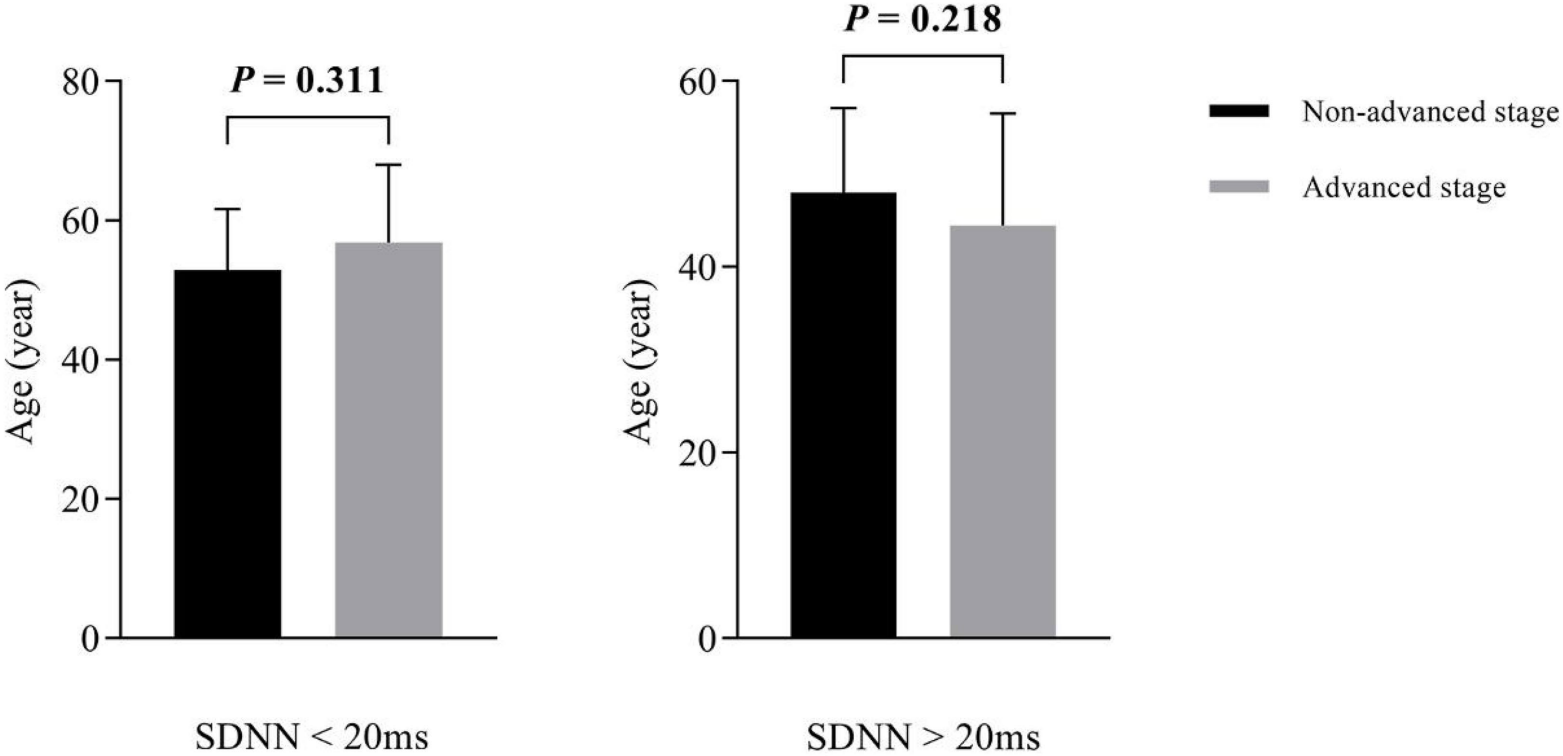
Subgroup analysis of age between non-advanced and advanced groups.

With SDNN=20ms as the cutoff value, the subjects were divided into two subgroups: a low-SDNN subgroup (SDNN<20ms; *n*=31) and a high-SDNN subgroup (SDNN>20ms; *n*=102). There was no statistically significant difference in age between the non-advanced and advanced groups (low-SDNN subgroup, *p*=0.311; high-SDNN subgroup, *p*=0.218; Figure 1).

To test the independent contribution of HRV to breast tumor stage, we performed multiple logistic regression models after adjusting for age, BMI, mean HR, and RR (Table 4). The associations of tumor stage with SD2, SDNN, VLF, TP, and LF were significant in logistic regression analysis. Specifically, for each 1-SD increase in SD2, SDNN, VLF, TP, and LF, the odds of having advanced staging decreased by 69.3% [odds ratio (OR): 0.307, 95% CI: (0.155, 0.606)], 64.3% [OR: 0.357, 95% CI: (0.183, 0.694)], 58.3% [OR: 0.417, 95% CI: (0.216, 0.807)], 53.3% [OR: 0.467, 95% CI: (0.239, 0.910)], and 65.9% [OR: 0.341, 95% CI: (0.130, 0.898)], respectively. The associations of tumor stage with CD, RMSSD, SD1, and HF were not significant in the logistic regression models.

**Table 4:** Results from Logistic regression models (adjusted for age, BMI, Mean HR, and RR).

Variables	OR	95% CI for OR		*p*
		Lower	Upper	
SD2 (ms)	0.307	0.155	0.606	**0.001**
SDNN (ms)	0.357	0.183	0.694	**0.002**
VLF (ms^2^)	0.417	0.216	0.807	**0.009**
TP (ms^2^)	0.467	0.239	0.910	**0.025**
LF (ms^2^)	0.341	0.130	0.898	**0.029**
CD	0.599	0.326	1.099	0.098
RMSSD (ms)	0.848	0.492	1.462	0.553
SD1 (ms)	0.869	0.505	1.495	0.612
HF (ms^2^)	1.000	0.632	1.583	1.000

BMI, body mass index; CI, confidence interval; CD, correlation dimension; HF, high-frequency power; HR, heart rate; LF, low-frequency power; OR, odds ratio; RMSSD, root mean square of successive interval differences; RR, respiration rate; SD, standard deviation; SDNN, standard deviation of all normal-to-normal intervals; TP, total power; and VLF, very low frequency. Bold values of *p* indicate statistical significance (value of *p*<0.05).

## Discussion

This study aimed to compare the HRV of patients with breast tumors (benign tumors, early-stage BC, and advanced-stage BC) and evaluate the feasibility of HRV as a tool for the early diagnosis and prognosis of BC patients. Our results revealed that patients with advanced-stage BC had lower HRV than those with benign tumors and early-stage BC. However, no statistically significant difference was observed in the HRV indices between the benign and early-stage groups. After adjusting for age, BMI, Mean HR, and RR, our results showed that SD2, SDNN, VLF, TP, and LF were associated with tumor stage.

The vagus nerve also called the wandering nerve, works *via* many neurotransmitters and plays an important role in multiple systems, such as cardiovascular, neuroendocrine, and immunological (Tracey, 2009). Studies have showed that the vagal nerve system transmits a variety of signals to the brain in order to restore the body to a steady state (Tracey, 2009; Ohira et al., 2013). Moreover, published preliminary studies have also demonstrated that vagal nerve tone is vital in the prognosis of cancer. A higher vagal nerve tone may protect cancer patients by reducing inflammation (De Couck et al., 2016). Measuring the HRV is a noninvasive approach of measuring vagal nerve activity (Vanderlei et al., 2009). HRV is known to be associated with vagal nerve tone (*r*=0.88; Kuo et al., 2005). In recent years, various linear and nonlinear methods have been applied to analyze the time series of heartbeat cardiac intervals, reflecting the physiological and pathological information contained in the HRV signal from different angles.

Few studies have explored the association of HRV with TNM in patients with breast cancer. In one study, Mouton et al. (2012) examined the data of 72 patients with colorectal cancer and found that the baseline HRV could predict the carcinoembryonic antigen levels at 12months. Moreover, they found that SDNN<20ms was associated with significantly higher CEA at 12months. Arab et al. (2016) showed the clinical significance of HRV in patients with breast cancer.

Several studies indicated that higher resting HF was strongly associated with longer overall survival in patients with recurrent or metastatic BC (Giese-Davis et al., 2015). Chiang et al. (2010) found that the survival time of patients with terminal hepatocellular carcinoma was significantly related to HF. More importantly, the results of these studies also indicated that HF strongly positively correlated with prognosis, particularly in patients with advanced-stage cancer. However, despite the interesting results of our study, we did not find any statistically significant differences in the HF among the three cancer stage groups. The difference in mean HR and RR may cause HF to not accurately estimate the cardiac autonomic regulation activity. In our study, the difference was not statistically significant in mean HR and RR between the groups, and the RR of all the enrolled subjects were in the HF band. Therefore, the interpretation of spectral HRV indices as autonomic cardiac regulation markers in our study was more accurate and reliable. The findings of the present study could be verified through a prospective study performed over a longer follow-up period.

Similar to the above results, the median HF at baseline was 122 (IQR, 55, 255) ms^2^ in our study, and the median HF values in benign, early-stage, and advanced-stage BC were 151 (78, 300), 151 (55, 270), and 60 (23, 136), respectively, indicating that a higher HF may be associated with benign or early-stage BC, and consequently a better survival rate. However, unlike Mouton et al. (2012), we used a cutoff of SDNN=20ms, and found that the SDNN showed a significant inverse association with tumor stage. A lower SDNN was found to be associated with more advanced-stage BC.

The interpretation of LF/HF is controversial. Malliani et al. (1991) showed that the LF/HF could reflect the balance between the vagus nerve and the sympathetic nerve. But LF/HF has been largely criticized as a marker of sympatho-vagal balance (Billman, 2011, 2013). First, LF power is not a pure index of sympathetic nerve, it may also be affected by vagus nerve and other unspecified factors. Second, sympathetic and vagus nerve can be simultaneously active, and their interaction is complex and nonlinear. Third, respiratory parameters and mechanical factors will also cause uncertainty in the contribution of sympathetic and vagus nerve to LF/HF. Finally, HR can affect LF/HF independently of cardiac autonomic nerve activity (Billman, 2013). In our study, no statistically significant difference was observed in LF/HF among the three cancer stage groups. This could be because of the complex physiological basis of LF/HF and the other unidentified factors.

Linear methods cannot be used to describe properly the complex nonlinear behavior, which is predominant in human systems. Therefore, it is necessary to search the novel indexes to reflect the correlation and the complexity characteristics of the HRV signal. The characteristic of HRV nonlinear analysis can better express the irregularity, complexity, and other dynamic characteristics of heartbeat fluctuations. Across various studies in the field of cardiovascular disorders, nonlinear dynamical HRV analysis is significantly superior to linear time-domain and frequency-domain methods (Mäkikallio et al., 2001; Stein et al., 2005; Voss et al., 2009; de Godoy, 2016). Some preliminary studies also explored the correlation between cancer and several nonlinear heartbeat dynamics measurements. Bettermann et al. (2001) showed that the variability, complexity, or rhythmicity of HRV in patients with BC was lower than that in patients with diabetes and age-matched healthy women. In particular, while comparing patients with and without BC metastasis, patients with metastasis had lower ApEn compared with those without metastasis. Shi et al. (2019) explored the perturbations of HRV nonlinear dynamical patterns to predict the increase in the severity of gastric cancer and found that nonlinear HRV parameters were the markers of autonomic nervous function to tumor progression.

Although, analysis of HRV by methods based on nonlinear dynamics do not reflect vagal or sympathetic regulation, we found significant correlations between time- and frequency domain indices and some of the nonlinear HRV parameters in patients with BC. For example, SampEn, α_1_, and CD correlated with RMSSD [SampEn (*r*=0.546, *p*<0.001), α_1_ (*r*=−0.564, *p*<0.001), and CD (*r*=0.800, *p*<0.001)], and also correlated with HF [SampEn (*r*=0.521, *p*<0.001), α_1_ (*r*=−0.530, *p*<0.001), and CD (*r*=0.792, *p*<0.001)]. This illustrates that the nonlinear parameters also contain the component of time-frequency domain index. The nonlinear analysis method is still in the preliminary exploration stage, and the exact physiological and pathological background has not been fully clarified. The findings of the current study might provide new evidence on the role of nonlinear HRV in cancer. Further studies are needed to clarify the correlation of nonlinear HRV as a long-term predictor of survival.

This study had some limitations. First, its cross-sectional study design was a major notable limitation. The correlation between HRV parameters and outcomes could not be inferred. Second, a comprehensive understanding of the connections of nonlinear HRV with BC prognosis is currently lacking. Third, more background variables, such as physical activity, stress levels, use of medications, and other relevant medical variables, could not be included. To address these limitations, studies with larger sample sizes, more detailed background variables, and a prospective design should be conducted to clarify the correlation of linear and nonlinear HRV parameters with BC prognosis.

## Conclusion

This novel study investigated linear and nonlinear HRV parameters in breast tumor groups. It found that the HRV was related to BC staging, indicating a correlation between tumor and HRV. The results of our study showed that patients with advanced-stage BC had lower HRV and might have a poor prognosis, and demonstrated that nonlinear HRV parameters might predict tumor staging in patients with breast tumors. Nonlinear approaches are of great significance in coping well with the nonstationary and nonlinear nature of heartbeat fluctuations. It is suggested that the combined measurement of linear and nonlinear HRV parameters may benefit future investigations. Researchers should identify a comprehensive biomarker for predicting BC prognosis by leveraging existing linear methods and nonlinear indicators. In addition to evaluating tumor stage, vagal nerve activity should be considered to estimate the prognosis of a cancer patient. Vagal nerve activity can be easily assessed and has the potential to provide healthcare professionals with incremental information based on the treatment plans. Future research should investigate the therapeutic potential of vagal nerve activation in cancer treatments through different supportive therapies such as relaxation, exercise interventions, and Traditional Chinese Medicine treatments (Niederer et al., 2013).

## Data Availability Statement

The original contributions presented in the study are included in the article/supplementary material, further inquiries can be directed to the corresponding authors.

## Ethics Statement

This study followed the regulations of the National Research Ethics Committee and obtained the approval of the Clinical Medical Research Ethics Committee of the First Affiliated Hospital of Bengbu Medical College, China. The patients/participants provided their written informed consent to participate in this study.

## Author Contributions

BS: conceptualization, resource allocation, and review and editing of the manuscript. SW and MC: data collection, interpretation of the results, and manuscript preparation. JW: data analysis. YZ: supervision and resource allocation. All authors contributed to the article and approved the submitted version.

## Funding

This study was funded by the “512” Outstanding Talents Fostering Project of Bengbu Medical College under Grant BY51201312, the Scientific Research Innovation Project of Bengbu Medical College under Grant BYKC201905, and the Students Scientific Research Innovation Project of Bengbu Medical College under Grant byycx20103.

## Conflict of Interest

A direct family member of BS owns stock in HeaLink Ltd., Bengbu, China.

The remaining authors declare that the research was conducted in the absence of any commercial or financial relationships that could be construed as a potential conflict of interest.

## Publisher’s Note

All claims expressed in this article are solely those of the authors and do not necessarily represent those of their affiliated organizations, or those of the publisher, the editors and the reviewers. Any product that may be evaluated in this article, or claim that may be made by its manufacturer, is not guaranteed or endorsed by the publisher.

## Footnotes

^1^ https://www.kubios.com
